# Structural and Functional Similarities between Osmotin from *Nicotiana Tabacum* Seeds and Human Adiponectin

**DOI:** 10.1371/journal.pone.0016690

**Published:** 2011-02-02

**Authors:** Marco Miele, Susan Costantini, Giovanni Colonna

**Affiliations:** 1 Department of Biochemistry and Biophysics and CRISCEB - (Interdepartmental Research Center for Computational and Biotechnological Sciences), Second University of Naples, Naples, Italy; 2 CROM (Oncology Research Centre of Mercogliano) “Fiorentino Lo Vuolo”, Mercogliano, Italy; Griffith University, Australia

## Abstract

Osmotin, a plant protein, specifically binds a seven transmembrane domain receptor-like protein to exert its biological activity via a RAS2/cAMP signaling pathway. The receptor protein is encoded in the gene ORE20/PHO36 and the mammalian homolog of PHO36 is a receptor for the human hormone adiponectin (ADIPOR1). Moreover it is known that the osmotin domain I can be overlapped to the β-barrel domain of adiponectin. Therefore, these observations and some already existing structural and biological data open a window on a possible use of the osmotin or of its derivative as adiponectin agonist. We have modelled the three-dimensional structure of the adiponectin trimer (ADIPOQ), and two ADIPOR1 and PHO36 receptors. Moreover, we have also modelled the following complexes: ADIPOQ/ADIPOR1, osmotin/PHO36 and osmotin/ADIPOR1. We have then shown the structural determinants of these interactions and their physico-chemical features and analyzed the related interaction residues involved in the formation of the complexes. The stability of the modelled structures and their complexes was always evaluated and controlled by molecular dynamics. On the basis of these results a 9 residues osmotin peptide was selected and its interaction with ADIPOR1 and PHO36 was modelled and analysed in term of energetic stability by molecular dynamics. To confirm *in vivo* the molecular modelling data, osmotin has been purified from *nicotiana tabacum* seeds and its nine residues peptide synthesized. We have used cultured human synovial fibroblasts that respond to adiponectin by increasing the expression of IL-6, TNF-alpha and IL-1beta via ADIPOR1. The biological effect on fibroblasts of osmotin and its peptide derivative has been found similar to that of adiponectin confirming the results found *in silico*.

## Introduction

Osmotin is a vegetable protein belonging to the pathogenesis-related (PR)-5 family of plant defense proteins that induces apoptosis in the yeast [1]. It has been found in many fruits, seeds and vegetables such as grapes, oats and tomatoes where it plays an antifungal activity [2]. It induces a programmed cell death in *saccaromyces cerevisiae* through RAS2/cAMP [3]. Yun et al. have shown that changes in the yeast cell wall that enhance toxicity are induced by osmotin via activation of a mitogen-activated protein kinase Cascade [4]. The osmotin protein has a specific receptor membrane encoded by the gene ORE20/PHO36 (YOL002c) coding for a seven transmembrane domain receptor-like protein [2]. In particular, the product of ORE20/PHO36 specifically binds osmotin at the plasma membrane and controls osmotin-induced cell death via a *RAS2* signaling pathway [2]. These observations open interesting questions since the mammalian homolog of PHO36 is a receptor for the human hormone adiponectin (ADIPOQ). What is surprising is that osmotin and adiponectin, the receptor binding proteins, do not share sequence similarity even if both have a similar internal beta-barrel domain [5].

However, what is more interesting is that even osmotin can induce AMP kinase phosphorylation in mammalian C2C12 myocytes via adiponectin receptors. These experimental observations suggest that osmotin binds the adiponectin receptor (ADIPOR1) in a cellular environment, made up of human cells, by activating the same signaling path of adiponectin [2]. If so, osmotin could be a polypeptide adiponectin-like with molecular and functional mechanisms similar to those exercised by human hormone.

On the basis of this hypothesis we have searched answers to the following questions: a) What are the structural similarities in the interaction of osmotin and adiponectin with the adiponectin receptor (ADIPOR1)? b) Knowing the structural basis of their interaction with the receptor, is it possible to isolate a biologically active peptide which mimes the adiponectin binding to ADIPOR1? We have used some methods of molecular modeling for searching and having those structural answers able to address functionally our questions. Therefore, in this paper we report the 3D modeling of the globular domain of the human adiponectin trimer (ADIPOQ), that of its receptor, ADIPOR1, that binds the globular domain, and that of PHO36. Moreover, we also modeled the ADIPOQ/ADIPOR1, osmotin/PHO36 and osmotin/ADIPOR1 complexes. Then, all the amino acids at the interface in these complexes were evaluated in order to know the structural and chemical features of these interaction residues that might be useful in the drug design involved in the treatment of obesity-related insulin resistance. Therefore, we have selected a small osmotin peptide (peptide^OSM^) and evaluated its ability to interact with ADIPOR1 and PHO36. The peptide^OSM^/ADIPOR1 complex shows that the peptide is firmly positioned in the same area of receptor with which both the adiponectin and the osmotin interact. Finally, experiments on synovial fibroblasts have shown that this peptide, as well as the whole osmotin, interacts with ADIPOR1 by activating the same signaling pathway activated by the adiponectin. The results, even if at an initial stage, show that the peptide^OSM^ can be taken into account as adiponectin-receptor agonist, once suitably amended to make it enzymes resistant.

## Methods

### Modelling of human adiponectin trimer

The three-dimensional model of the three human adiponectin chains (UniProt code: Q15848, region 108–244) was performed by a comparative modelling strategy using as template structures the related mouse adiponectin chains (PDB code: 1C3H). Since the template structure contained two trimers having different loop conformations among beta-strands, we have used: i) the murine A and D chains to model the human monomer A, ii) the murine B and E chains as template for the human monomer B and iii) the murine C and F chains as template for the human monomer C. The sequence alignment was made by using CLUSTALW program [6]. The MODELLER9 v5 program [7]–[8] was used to build 10 full-atom models of the three adiponectin chains by setting 4.0 Å as root mean square deviation (RMSD) among initial models and by full model optimization. To select the best models, we used the ProsaII program [9] to check the fitness of the sequences relative to the obtained structures and to assign a scoring function, and the PROCHECK program [10] to evaluate their stereochemical and structural packing quality. Cysteine side-chains refinement was performed by SCAP module of Jackal package [11]. Secondary structures were assigned by the DSSP program [12]. Search for structural classification was performed on the CATH database [13]. Secondary structure predictions were performed by Jpred server [14]. The structure of the human adiponectin trimer was assembled by superimposing the three best modelled chains of human monomers to the corresponding murine chains, to obtain the same relative orientation of the three subunits. This model was minimized by using 500 steps of energy minimization under conjugate gradient algorithm in order to optimize side chain conformations and avoid sterical clashes according to the commonly used procedure [15]–[19]. The “Protein–Protein Interaction Server” [20] and NACCESS program [21] were used to identify the amino acids at the interface and to evaluate their solvent accessibility. The presence of putative H-bonds was calculated with Hbplus program [22] but the putative salt-bridges were evaluated using the program recently developed by our group [23]. We have also tried to model adiponectin-examers and higher polymers by docking methods because in PDB there are not structures that can be used as template but only the trimer of mouse adiponectin. However attempts were unsuccessful from a modeling point of view because the models were not so stable during the molecular dynamics experiments. Since x-ray crystallographic studies [2] have shown that both proteins share the same lectin-like domain, as well as the structurally active amino acids which are present on the outer surface of the adiponectin trimer, we have decided to use the trimer in our studies. Moreover, we have modeled only the globular part of ADIPOQ (without the related collagen part) because our aim was to model its complex with ADIPOR1, receptor for the globular part.

### Modelling of human ADIPOR1 and PHO36

Modeling of the human adiponectin receptor 1 (ADIPOR1), (UniProt code: Q96A54, region 135–355) was obtained using an integrated protocol based on the method of fold-recognition and comparative modeling and already used in our recent paper [24]. In particular, we have searched accurately the template for our modeling procedure using a database of structurally characterized families of membrane proteins with 7 helix topology (http://opm.phar.umich.edu/) as well as a fold recognition program LOOPP [25]. In this case the solution of fold recognition problems follows from membrane localization of the receptor and reliable prediction of 7 membrane alpha-helices. Our research has evidenced that the structure of archeal (*Natronomonas pharaonis*) phototaxis protein rhodopsin II (PDB code: 1GU8) presented similar lengths of helices and loops between the helices respect to ADIPOR1 and, thus, suggested that this protein was the correct template for predicting the three-dimensional structure of ADIPOR1 by comparative modelling strategy. Moreover, in the obtained model the loop regions were refined using the LOOPY module of Jackal package, that appeared to yield the best results in loop modelling, with models that are on average of 2–8% better than those generated by other programs [26]. We have also modeled yeast PHO36 (UniProt code: Q12442, region: 80–300) using the same procedure described for human ADIPOR1 and as template the structure of archeal (*Natronomonas pharaonis*) phototaxis protein rhodopsin II (PDB code: 1GU8).

### Modelling of ADIPOQ/ADIPOR1 and Osmotin/ADIPOR1 complexes

Firstly PatchDock web server [27] was used to model the ADIPOQ/ADIPOR1 and Osmotin/ADIPOR1 complexes using the obtained models for ADIPOQ and ADIPOR1 by the comparative modelling strategy and the crystallographic structure of Osmotin (PDB code: 1PCV) [5]. This program is a geometry-based molecular docking algorithm aimed at finding docking transformations that yield good molecular complementary shape [28]. Then, flexible refinement and re-ranking of the rigid docking solution candidates was performed by FiberDock, that accounts for both backbone and side-chain flexibility [29]. In particular, this method optimizes the side chain conformations in the interface, models backbone movements, minimizes the relative orientations of the molecules and ranks the refined solutions by a binding energy scoring function [29]. We have chosen FiberDock because it has been used in last CAPRI experiment up to 40 targets and its flexible refinement process resulted in a drastic improvement in the accuracy of predicted complexes [30]. The complexes were analyzed by the same programs used for the adiponectin trimers and in other our recent papers [15]–[19].

### Modelling of an osmotin peptide and of its complex with ADIPOR1

Starting from the osmotin structure we have selected the peptide CTQGPCGPT, (segment 157–165), that hereafter we will name as Peptide^OSM^. The peptide shows a disulphide bond between Cys 157 and Cys 162 that stabilizes it during the simulations. This structural feature proved to be important in comparison with all other different peptides screened from osmotin as well as the same adiponectin and involved in the interaction surface between osmotin/adiponectin and ADIPOR1. These peptides were poor ligands from an energetic point of view probably because of their high flexibilities. The chosen peptide was subjected to molecular dynamics by GROMACS software package (v3.3.1) [31] in cubic boxes filled with SPC216 water molecules and GROMOS43a1 was selected as force-field. To optimize the system, the peptide model was previously subjected to energy minimization and positional restraints cycles. The simulation was carried out with periodic boundary conditions by adding sodium ions in order to have a value of zero as net electrostatic charge of the system. The bond lengths were constrained by the all atoms LINCS algorithm. Particle Mesh Ewald (PME) algorithm was used for the electrostatic interactions with a cutoff of 0.9 nm, according to recent papers [19], [24], [32]. The simulations were performed at neutral pH with runs of 10 ns at room temperature (300 K) coupling the system to an external bath. GROMACS routines were utilized to check the trajectories and the quality of the simulations. The complex between Peptide^OSM^ and ADIPOR1 was modeled and analyzed using the same procedure reported above.

### Modelling of Osmotin/PHO36 and osmotin peptide/PHO36 complexes

Osmotin/PHO36 and Peptide^OSM^/PHO36 complexes were modeled and analyzed using the obtained model for PHO36 by comparative modelling strategy, the crystallographic structure of Osmotin (PDB code: 1PCV) [5] and the same procedures performed for the complexes with ADIPOR1 based on flexible refinement docking by FiberDock [29]–[30].

### Peptide^OSM^ synthesis

The solid-phase peptide synthesis by Fmoc chemistry was used to obtain our peptide (having pI = 5.50), combined with the S-acetamidomethyl protection for cysteines. Rink-amide resin was used as a solid-phase carrier, and TBTU was used as a coupling reagent. Piperidine in dimethylformamide (20% solution) was used to remove the Fmoc group at every step. The coupling and deprotection reactions were checked by the Kaiser test. Cleavage from resin and side-chain deprotection were achieved using a mixture of 82.5% TFA, 5% thioanisole, 5% m-cresol, 5% water, and 2.5% ethanedithiol). The peptide was analyzed on a reversed-phase high performance liquid chromatography (HPLC) C_18_ column and its purity was checked by MALDI-TOF mass measurements (Voyager-DE Biospectrometry). Disulfide bond was formed after cleavage and deprotection of the relevant linear peptide with Cys' protecting groups. The two side-chains protecting groups of cysteine were removed at the same time as peptide cleavage and the disulfide bond was formed using 0.95 equiv. of 2,20-dithiodipyridine (PySSPy) and 1 mM peptide in 0.1% (v/v) TFA in water at room temperature. The oxidation was completed after 2 h and the product was purified by preparative RP-HPLC. The product was identified by MALDI-TOF. The peptide was poorly soluble in water at low pH. Its solubility increased at higher pHs and at pH 6.5 was completely soluble. A batch solution 1×10^−3^ M Peptide^OSM^, 5×10^−3^ M K-phosphate buffer at pH 7.4 was used through the experiments diluting it each time to the appropriate concentration. The peptide concentration was determined by using a molar extinction coefficient at 276 nm of 165 M^−1^ cm^−1^ for Cistine as suggested by Pace et al. (1995) [33]

### Osmotin purification

Osmotin was purified to homogeneity from N*icotiana Tabacum* seeds. Purification was achieved by using a procedure consisting of an acid treatment step followed by two chromatography steps, already reported for an osmotin-like protein from the seeds of *Benincasa hispida* [34]. The protein concentration was determined by using a molar extinction coefficient at 280 nm of 36,650 M^−1^cm^−1^ calculated by the known content of Trp (5690 M^−1^cm^−1^), Tyr (1280 M^−1^cm^−1^ and Cys (120 M^−1^cm^−1^) present in the sequence [33]. The protein purity was assessed by SDS-PAGE and proteins stained with Coomassie brilliant blue.

### Cell culture

To elucidate *in vivo* the cellular effects of the osmotin and Peptide^OSM^ in comparison with those exerted by adiponectin, we have examined the IL6, TNF-alpha and IL-1beta profiles from normal Synovial tissues obtained by arthroscopic biopsy [35], [36]. Fresh tissues were minced and digested in a solution of collagenase and DNase. Isolated fibroblasts were filtered. The cells were grown on the plastic cell culture dishes in 90% air-10% CO2 in Dulbecco's modified Eagle's medium, high glucose, supplemented with 10% HI-FBS, 2 mmol/L l-glutamine, 10 µmol/L nonessential amino acids (Life Technologies, Inc.), 100 U/ml penicillin, and 100 µg/ml streptomycin sulfate. The purity of the isolated cells was assessed by flow cytometry (FACS). Fibroblasts were cultured and expanded in vitro in monolayer. Fibroblasts from passages four to nine were used for the experiments.

### Evaluation of biological activity of adiponectin, osmotin and Peptide^OSM^

Human full-length adiponectin (Catalog no.1065-AP; R&D Systems) (5 µg/ml), osmotin (5 µg/ml) and peptide (5 µg/ml) administration was followed by 24 h of incubation at 37°C in a humidified incubator and separate plates, the media were then removed and stored at −80°C until assay. IL-6, TNF-alpha and IL-1beta in the medium were assayed using a multiplex biometric ELISA-based immunoassay, containing dyed microspheres conjugated with a monoclonal antibody specific for a target protein and a procedures already used in our previous article [37]. In particular, the cytokine concentrations of IL6, TNF-alpha and IL-1beta were measured using a 27-Plex panel (Bioplex, Bio-Rad Lab., Inc., Hercules, CA, USA) able to measure at the same time all the cytokines. Each reaction was done in triplicate. Briefly, 30 µl of medium sample was diluted 1∶4 and incubated with antibody-coupled beads. Complexes were washed, then incubated with biotinylated detection antibody, and, finally, with streptavidin-phycoerythrin prior to assessing cytokine concentration titers. Cytokine levels were determined using a Bio-Plex array reader (Luminex, Austin, TX).

## Results

### Human adiponectin model

**Figure 1:**
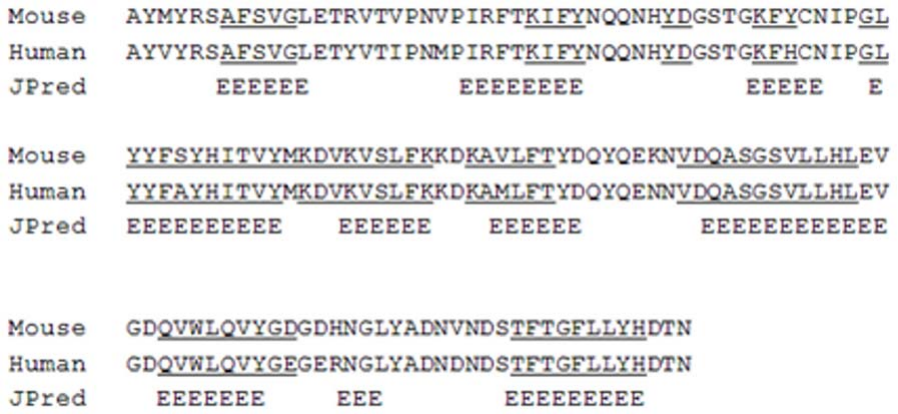
Alignment of adiponectin monomer A in mouse and human. Secondary structure prediction made by JPred. Amino acids in the beta-strands are underlined.

**Figure 2:**
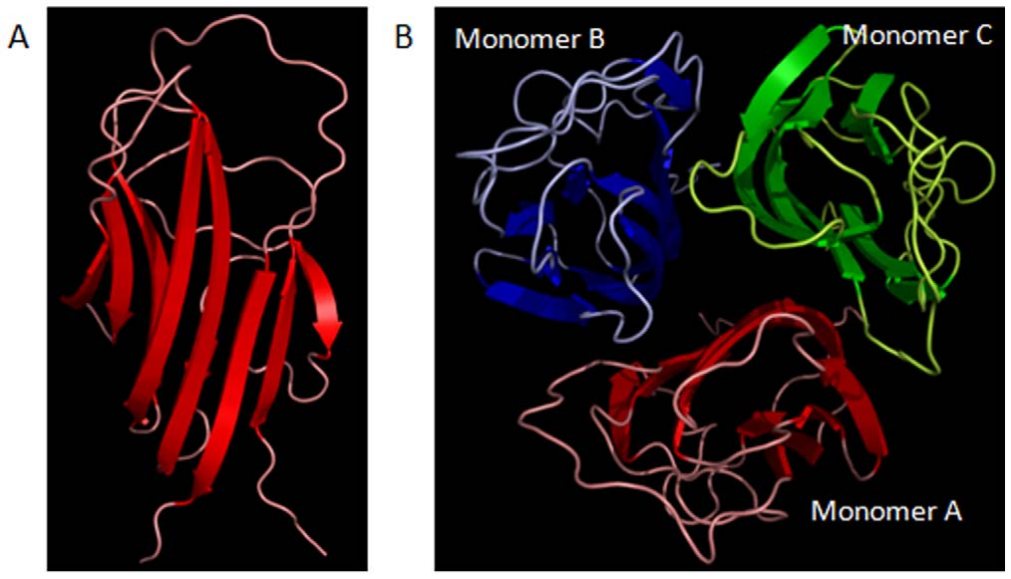
3D model of monomer A and trimer of human adiponectin. The beta-strands are indicated with arrows.

The three-dimensional model of the human adiponectin trimer was performed, according to the comparative modelling strategy and using as template structures three murine chains because the sequence identity percentage between human and mouse proteins was equal to 91%. Figure 1 shows the alignment of an adiponectin monomer in human and mouse. Starting from this alignment, a set of 10 full-atoms models was generated for each human monomer. The best models (see Table S1) had Prosa Z-score of −4.7 and 92% of residues were in most favored regions. These values, compared with those of the template murine structures, indicated the good quality of models. Secondary structure predictions made by JPred program agree well with the adiponectin models obtained by comparative modeling. The adiponectin monomer (shown in Figure 2A) present 11 beta-strands organized in an architecture of “mainly beta sandwich” type. Moreover, the models of human and mouse adiponectin were compared by structural superimposition as well as in terms of secondary structures. RMSD values resulted in about 0.25 Å each. Moreover, the comparison of the secondary structures evidenced that beta-strands were well conserved along the sequence, with very few changes. In Figure 2B the model of adiponectin trimer is shown. The three monomers presented very similar conformations in terms of secondary structures as in the mouse trimer (PDB code: 1C3H). In particular, the location and length of beta-strands were equal in the three monomers. We found very small differences located only in loop conformations and a mean RMSD value of 0.17 Å was obtained by structural superimposition of the three human monomers. We have also evaluated the interactions features between the monomers in two human and mouse trimers (see Table S2). These complexes show small differences in the values of the interface areas, and in the number of interaction residues, of H-bonds and salt-bridges that depend on few not conserved residues in two mammalian sequences.

### Human ADIPOR1 model

**Figure 3:**
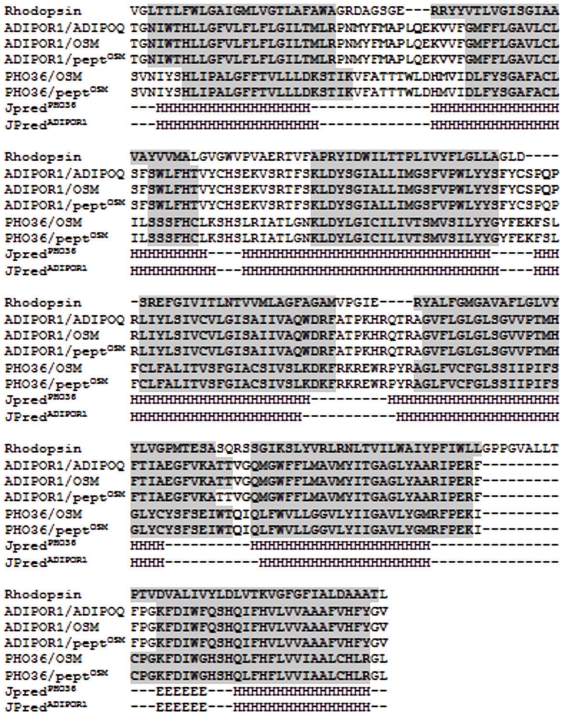
Alignment of Rhodopsin, ADIPOR1 and PHO36. Secondary structure predictions made by JPred are reported for ADIPOR1 and PHO36. Amino acids in the helices are reported in grey but those interacting with the ADIPOQ, osmotin (OSM) and Peptide^OSM^ in the related complexes with ADIPOR1 and PHO36are evidenced in bold.

**Figure 4:**
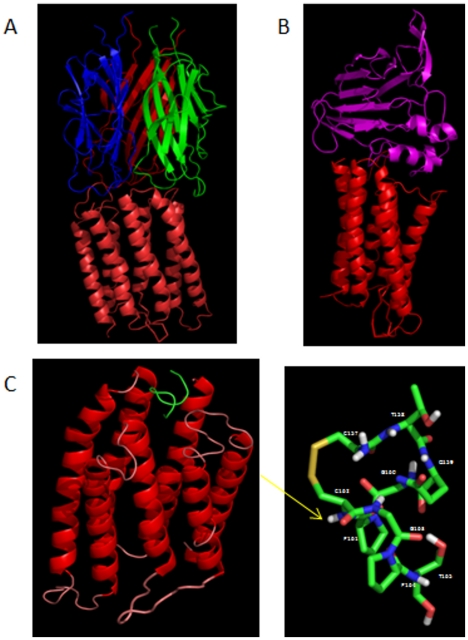
3D model of three complexes. (A) AdipoQ/ADIPOR1 complex, (B) Osmotin/ADIPOR1 complex and (C) PeptideOSM/ADIPOR1 complex with a zoom on the Peptide^OSM^ structure.

We have modeled ADIPOR1 receptor because it is known to bind the globular part of ADIPOQ (i.e. without the related collagen part). The alignment between ADIPOR1 and the template structure used for the modelling procedure is reported in Figure 3. The obtained model shows Prosa Z-score of −2.97 and 93.2% of residues in the most favored regions. These values, compared with those of the template structure, indicate that our model has a good energetic and stereo-chemical quality as we have also found in the adiponectin model. Secondary structure predictions made by JPRED agree with the obtained structure by comparative modelling. ADIPOR1 model is composed by seven trans-membrane helices and several intra- and extra-cellular loops characteristics of all the transmembrane receptors (Figure 4). Comparing the secondary structures between ADIPOR1 and rhodopsin models it is evident that the seven transmembrane helices are well conserved with only few changes in helix lengths (see Figure 3).

### PHO36 model

Figure 3 shows the alignment between PHO36 and the template structure used for the modelling procedure. The obtained model shows Prosa Z-score of −2.4 and 95.9% of residues in the most favored regions. It is composed by seven trans-membrane helices and several intra- and extra-cellular loops characteristics of all the transmembrane receptors as well as for ADIPOR1 (Figure S1). Comparing the secondary structures of PHO36, ADIPOR1 and rhodopsin models it is evident that the seven transmembrane helices are well conserved with only few changes in helix lengths (see Figure 3). Moreover, the models of human ADIPOR1 and yeast PHO36 were also compared by structural superimposition and an RMSD value of 0.24 Å confirmed that these two receptors had very similar structures.

### Modelling of ADIPOQ/ADIPOR1 and Osmotin/ADIPOR1 complexes

The ADIPOQ/ADIPOR1 complex was modeled (Figure 4A) and the interaction between the adiponectin and receptor regions were analyzed in terms of surface (ASA) and residues to the interface, H-Bonds, salt bridges (Tables 1 and 2). The adiponectin interacts with ADIPOR1 by the loops at the top of the globular domain and ADIPOR1 by its extracellular loops. In the best obtained complex the ADIPOQ and ADIPOR1 chains form 3 H-bonds and 15 salt bridges. Instead, in the osmotin/ADIPOR1 complex (Figure 4B) the osmotin interacts with ADIPOR1 by 3 H-bonds and 14 salt bridges. In particular, the osmotin exposes the amino acids located in beta-strands, helices and loop (Figure S2) while ADIPOR1 interacts by its extracellular loops as already seen in the ADIPOQ/ADIPOR1 complex.

**Table 1:** Analysis of the ADIPOQ/ADIPOR1, Osmotin/ADIPOR1, Peptide^OSM^/ADIPOR1, Osmotin/PHO36, and Peptide^OSM^/PHO36 complexes in terms of interface surface area (Å^2^), number of interaction residues, interchain H-bonds and salt bridges evaluated for each chain.

Chains	Interface surface area	Number of interaction residues	Interchain H-bonds	Salt brigdes
**ADIPOQ (Chain A)**	207.1	7	1	4
**ADIPOR1**	220.48	9	1	4
				
**ADIPOQ (Chain B)**	645.45	18	1	5
**ADIPOR1**	735.89	14	1	5
				
**ADIPOQ (Chain C)**	619.43	16	1	6
**ADIPOR1**	676.42	10	1	6
				
**Osmotin**	1068.6	26	3	14
**ADIPOR1**	1072.92	18	3	14
				
**Peptide^OSM^**	497.46	9	3	4
**ADIPOR1**	388.04	10	3	4
				
**Osmotin**	683.72	21	3	5
**PHO36**	679.54	17	3	5
				
**Peptide^OSM^**	345.06	9	2	2
**PHO36**	304.38	8	2	2

**Table 2:** List of interaction residues in ADIPOQ/ADIPOR1, Osmotin/ADIPOR1, Peptide^OSM^/ADIPOR1, Osmotin/PHO36, and Peptide^OSM^/PHO36 complexes.

Peptide	Residue
**ADIPOQ (Chain A)**	**Asp** **170**, Tyr 186, Gln 188, Tyr 189, Gln 190, **Glu 191**, Asn 192
**ADIPOQ (Chain B)**	**Lys** **169**, **Asp** **170**, Tyr 186, **Asp** **187**, Gln 188, Tyr 189, Gln 190, **Glu** **191**, Asn 192, Asn 193, **Glu** **218**, **Arg** **221**, Asn 222, Gly 223, Leu 224, Tyr 225, Ala 226, **Asp** **227**
**ADIPOQ (Chain C)**	Tyr 167, **Lys** **169**, **Asp** **170**, Tyr 186, Gln 188, Tyr 189, Gln 190, **Glu** **191**, Asn 192, Asn 193, Val 194, **Glu** **218**, **Arg** **221**, Gly 223, Leu 224, Ala 226
**ADIPOR1**	Met 161, Tyr 162, Phe 163, Met 164, Ala 165, Pro 166, Leu 167, Gln 168, **Lys** **170**, Val 171, Phe 228, Tyr 229, Cys 230, Ser 231, Gln 233, Pro 234, **Arg** **235**, Val 297, Gly 298, Gly 354, Val 355
**Osmotin**	Gly 118, **Lys 119**, Cys 157, Thr 158, Gln 159, Gly 160, Pro 161, Cys 162, Gly 163, Pro 164, Thr 165, Phe 166, **Lys** **169**, **Lys** **172**, Gln 173, **Asp** **177**, Tyr 179, Tyr 181, Pro 182, **Asp** **184**, **Asp** **185**, Pro 186, Thr 187, Thr 189, Phe 190, Thr 191
**ADIPOR1**	Tyr 162, Met 164, Ala 165, Pro 166, Leu 167, **Lys** **170**, Val 171, Phe 228, Tyr 229, Cys 230, Ser 231, Gln 233, Pro 234, **Arg** **235**, Val 297, Gly 298, Gly 354, Val 355
**Peptide^OSM^**	Cys 157, Thr 158, Gln 159, Gly 160, Pro 161, Cys 162, Gly 163, Pro 164, Thr 165
**ADIPOR1**	Tyr 162, Met 164, Ala 165, Pro 166, **Lys 170**, Val 171, Phe 228, Val 297, Gly 298, Val 355
**Osmotin**	Phe 150 **,** Gly 151**,** Gln 153**,** Gln 154**,** Tyr 155 **,** Cys 157**,** Thr 158**,** Gln 159**,** Gly 160**,** Pro 161**,** Cys 162**,** Gly 163**,** Pro 164**,** Thr 165**,** Phe 166 **,** **Lys** **169,** Tyr 181 **,** Gln 183**,** **Asp 184**, **Asp** **185,** Pro 186
**PHO36**	Ala 108**,** Thr 109**,** Thr 110**,** Trp 112 **,** His 115**,** Met 116**,** Val 117**,** Tyr 173 **,** Phe 174 **,** **Glu** **175,** **Lys** **176,** Phe 177 **,** Ser 178**,** Leu 179**,** Phe 180 **,** Gln 242**,** Ile 243
	
**Peptide^OSM^**	Cys 157**,** Thr 158**,** Gln 159**,** Gly 160**,** Pro 161**,** Cys 162**,** Gly 163**,** Pro 164**,** Thr 165,
**PHO36**	Thr 109, Thr 110**,** Thr 111**,** Trp 112 **,** Tyr 173 **,** **Lys** **176,** Gln 242**,** Ile 243

Charged amino acids are reported in bold while aromatics are underlined.

### Osmotin peptide model and its complex with ADIPOR1

**Figure 5:**
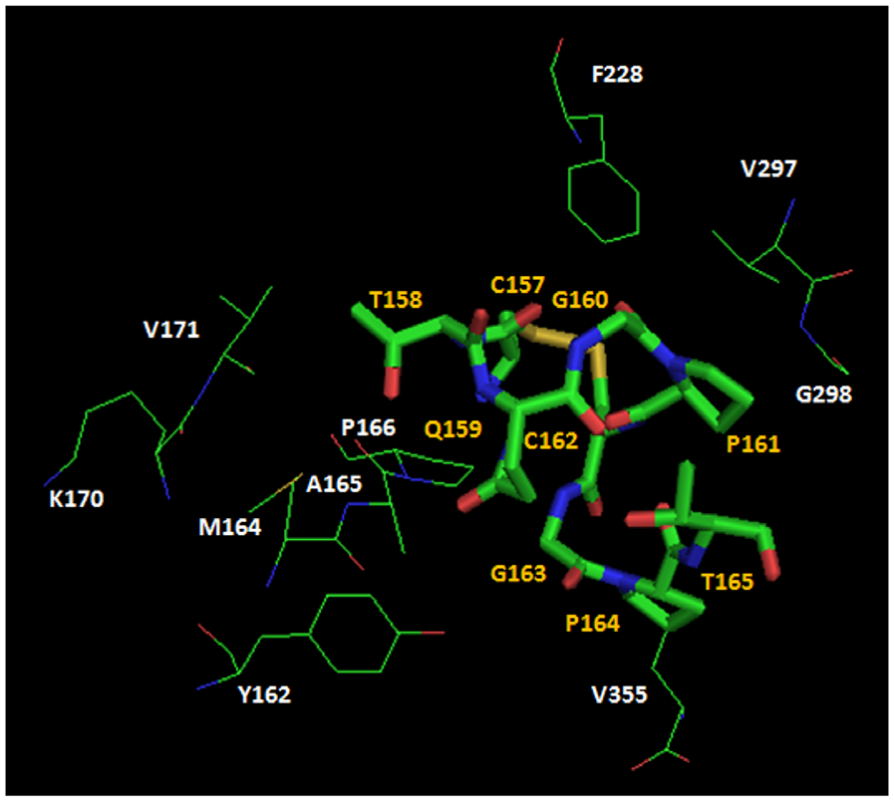
Details of Pept^OSM^ - ADIPOR1 interaction. The peptide is reported by stick representation and yellow labels. The interaction residues of ADIPOR1 are shown by lines and white labels.

The osmotin/ADIPOR1 complex presents a global architecture very similar to that shown by the adiponectin/ADIPOR1 complex (Figure 4A–B). The Peptide^OSM^ (region 157–165 in the osmotin sequence) is located at the interface between the osmotin and ADIPOR1 (Table 2). The possible fluctuations of its structure are reduced by the presence of the disulphide bond as demonstrated by the short molecular dynamics simulation time. In fact, a stable state was quickly reached after 3 ns and was constant up to the end of the dynamics (see Figure S3). In the Peptide^OSM^/ADIPOR1 complex the peptide has been found to interact with ADIPOR1 by 3 H-bonds and 4 salt bridge (Table 1 and Figures 4C and 5).

### Modelling of Osmotin/PHO36 and Peptide^OSM^/PHO36 complexes

The osmotin/PHO36 complex was modeled and evaluated in terms of surface (ASA) and residues to the interface, H-Bonds, salt bridges (Figure S1 and Tables 1 and 2). In this complex the osmotin interacts with PHO36 by 3 H-bonds and 5 salt bridges and exposes amino acids located in β-strands, helices and loop as found in the complex with ADIPOR1. In the Peptide^OSM^/PHO36 complex the peptide has been found to interact with PHO36 by 2 H-bonds and 2 salt bridges (Figure S1 and Tables 1 and 2).

### Biological activity of Osmotin and Peptide^OSM^

**Figure 6:**
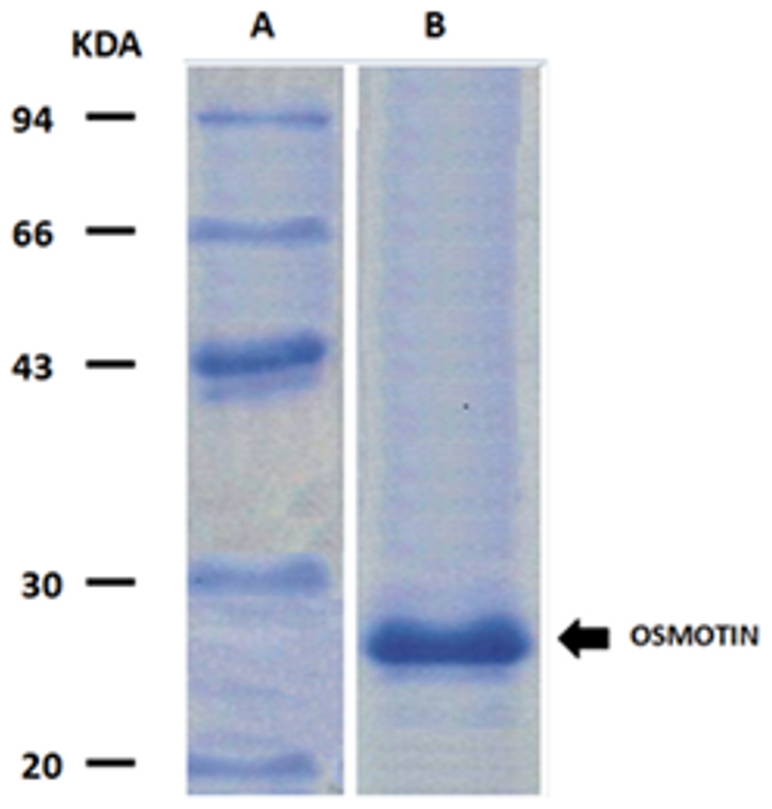
SDS-PAGE analysis of osmotin purified according to Shih et al. (2001) [34]. A. Molecular mass marker (LMW, Amersham Pharmacia Biotech); B. Protein is indicated by arrow. The gels were stained with Coomassie brilliant blue.

It has been recently demonstrated that adiponectin acts as an inducer of inflammatory response by increasing IL-6 production in human synovial fibroblasts. It was found that adiponectin increases the expression of IL-6 and ADIPOR1 but not ADIPOR2 [36]. In general, results suggest that ADIPOR1 is an upstream receptor in adiponectin-induced IL-6 release via the ADIPOR1 receptor/AMPK/p38/IKKαβ and NF-kB signaling pathway. Moreover, the same authors also found that the treatment with adiponectin caused TNF-alpha and IL-1beta release in human synovial fibroblasts [36]. Therefore, to explore the *in vivo* effect of osmotin and its derivative peptide on the adiponectin receptor ADIPOR1, we have purified osmotin from seeds of *Nicotiana Tabacum* at homogeneity according to Shih et al. (2001) [34]. In Figure 6 we show the SDS-PAGE of the purified fraction of osmotin used for our experiments. Then the Peptide^OSM^ was synthesized (see Methods) and its biological activity on IL-6, TNF-alpha and IL-1beta was evaluated in cultured synovial fibroblasts in comparison with adiponectin and whole osmotin. The results, reported in Table 3, clearly demonstrate that adiponectin as well as osmotin and Peptide^OSM^ show a biological activity raising the concentrations of the downstream signaling cytokines activated via ADIPOR1 as shown by Tang et al. (2007) [36]. Our results also confirm the work of Narashiman et al. 2005 [2] showing that osmotin interacts with ADIPOR1 in a manner similar to that utilized by adiponectin but even the Peptide^OSM^ shows a similar effect activating both the same receptor and the signaling pathway. We have also used two other peptides of nine and eleven residues, respectively, with random sequences but they did not show any biological activity in cultured synovial fibroblasts (*data not shown*). Even so two unrelated proteins (sperm whale apomyoglobin and bovine serum albumin (BSA)) were tested with negative results. It is noteworthy that BSA has been already used as a negative control to check the binding on unrelated proteins to osmotin receptor [2]. Furthermore, we also observed that the ability of osmotin to withstand the attack of cellular hydrolytic enzymes decreases slowly over time during the first 24 hours, as it becomes sufficiently rapid and substantial if the incubation continues over the 24 hours. The same is true for the peptide. This observation suggests that the Peptide^OSM^, even if biologically active, should be chemically modified to be used *in vivo* usefully.

**Table 3:** Effect of adiponectin, osmotin and peptide^osm^ on human synovial fibroblasts.

	basal	Adiponectin	Osmotin	Peptide^OSM^
**IL6 (pg/mL)**	32±6	110±16	95±16	89±16
**IL-1beta (pg/mL)**	62±11	163±18	141±18	130±18
**TNF-alpha (pg/mL)**	51±8	105±16	92±16	86±14

## Discussion

In this article, on the basis of experimental data of several authors [2], [5], we have executed molecular modeling studies on adiponectin and its receptor ADIPOR1, on osmotin, and on the various complexes possible among them with the aim to have a detailed knowledge of the interactions involved in the binding regions and to identify a peptide fragment to use in drug design as a possible putative adiponectin-receptor agonist. The results have enabled us to select a peptide fragment from osmotin of 9 amino acids. The peptide, rather rigid for the presence of a disulfide bond, showed a good affinity for the adiponectin receptor (ADIPOR1) in terms of binding energy. The indications obtained by modeling were put into practice by the synthesis of the peptide that was tested *in vivo* on cultured synovial cells together with osmotin. The biological response of the adiponectin-receptor was to activate the same signaling pathway enabled by the adiponectin hormone. Experimental data have demonstrated that the suppression of ADIPOR1 with small interfering RNA (siRNA) reduced the increase in fatty-acid oxidation by globular domain of the adiponectin [38]. Thus, the results regarding the expression or the suppression of ADIPOR1 support the conclusion that this protein serves as receptor for globular adiponectin and mediates increased AMPK, PPAR ligand activities and fatty-acid oxidation and glucose uptake by adiponectin. Moreover, a number of studies have shown that obesity and insulin resistance are accompanied by decreased adiponectin levels and that adiponectin replacement under experimental setting is able to diminish both insulin resistance and atherosclerosis. Therefore the adiponectin and its receptor represent possible therapeutic targets for the treatment of obesity-related insulin resistance. According to these data, a therapeutic strategy for type II diabetes, metabolic syndrome and cardiovascular disease may include the up-regulation of plasma adiponectin, the up-regulation of adiponectin receptors or the development of ADIPORs agonists able to stimulate adiponectin receptors [39].

Independent results showed that a protein from plants, with no sequence homology with the human adiponectin, interacts with a membrane receptor codified by the same type of gene encoding for the membrane receptor of human adiponectin [5]. Experimental results of Bressan et al. have also shown that the osmotin activates molecular mechanisms similar to those activated by the adiponectin binding to its receptor [2]. This makes osmotin a useful molecule to isolate a peptide that can be, in turn, used as a model to draw new molecules to be used as adiponectin receptor agonist. For these reasons we have modeled the complex between ADIPOQ and ADIPOR1 (Figure 4A) and analyzed also the physical-chemical properties of ADIPOQ and ADIPOR1 residues present in the interaction regions (Table 2). These two proteins expose both negatively and positively charged residues in ADIPOQ (i.e. Lys 169, Arg 221, Asp 170, Asp 187, Asp 227 and Glu 191, Glu 218) and two positively charged residues in ADIPOR1 (i.e. Lys 170 and Arg 235). These data suggest that the predominant interaction between the adiponectin and its receptor is on electrostatic basis. Furthermore, the presence of aromatic residues (i.e. Tyr 167, Tyr 186, Tye 189, Tyr 225 in ADIPOQ and Tyr 162, Tyr 229, Phe 163, Phe 228 in ADIPOR1) stabilizes the interaction between these two proteins and might play an important role to support the stacking interactions also with putative agonists and organic compounds. It is also known that osmotin, a tobacco PR-5 family protein, and adiponectin have similar structural folds but unrelated sequences [22]. Osmotin controls the apoptosis in yeast through a homolog of mammalian adiponectin receptor (PHO36) [2]. The human adiponectin receptor (ADIPOR1) and the one from yeast PHO36 present a sequence similarity of about 54% (Figure 3 and Figure S4). X-ray crystallographic studies revealed that the osmotin is composed of three domains of which domain I consists of 11 beta-strands, arranged in the shape of a beta-sandwich, domain II consists of several loops extending from domain I and stabilized by four disulfide bonds and domain III consists of a small loop with two disulfide bonds (Figure S5) [5]. In particular, the domain I overlaps with the adiponectin, suggesting that the two proteins share the lectin-like structural domain even if they do not show sequence similarity. Interestingly, it has been shown that the osmotin can activate AMP kinase in C2C12 myocytes [2] and the suppression of ADIPORs expression by siRNA markedly reduced phosphorylation of AMP kinase induced by osmotin. These data suggested that the osmotin activates AMP kinase via ADIPORs in mammalian C2C12 myocytes [2]. Whether the beneficial effects of adiponectin can be induced by osmotin is currently unknown. Therefore, we have also modeled the complex between osmotin and ADIPOR1 (Figure 4B). In this complex the osmotin exposes three positively charged residues (i.e. 3 Lys) and three negatively charged residues (i.e. 3 Asp) but ADIPOR1 exposes the same loop regions and the same charged residues evidenced in the ADIPOQ/ADIPOR1 complex (Table 2). Moreover, also in this complex the presence of aromatic residues (i.e. Tyr and Phe) stabilizes the interaction between these two proteins.

When the osmotin/ADIPOR1 and ADIPOQ/ADIPOR1 complexes are compared with superimposed ADIPOR1 receptors, the lectin domains of osmotin and of ADIPOQ are not in the same orientation (see Figure S6). Likely, this could depend from the different size of ADIPOQ trimer (about 400 residues) in respect to osmotin (205 residues). However, from the comparison between the two osmotin/ADIPOR1 and ADIPOQ/ADIPOR1 complexes, it is evident that the structural determinants of these interactions are very similar. In fact, the interaction regions of osmotin and ADIPOQ are almost completely superposed (see Figure S7), the presence of charged and aromatic residues at the interface is conserved in the two proteins, and the number of H-bonds with ADIPOR1 is exactly the same (Table 1). Also, both osmotin and ADIPOQ show a similar affinity for ADIPOR1 in terms of binding energy [40], as shown in Figure S8. Therefore, we think that all these evaluations indicate that osmotin monomer surface mimics ADIPOQ trimer surface in the context of receptor binding.

Comparing the Osmotin/PHO36 and Osmotin/ADIPOR1 complexes one can evidence that ADIPOR1 presents at the interface two positively charged residues (i.e. one Lys and one Arg) whereas PHO36 both positively (i.e. Lys) and negatively (i.e. Glu) charged residues. Moreover, the osmotin exposes a positively charged residue (Lys 169) and two negatively charged residues (Asp 184 and Asp 185) in the Osmotin/PHO36 complex, but at the interface in Osmotin/ADIPOR1 complex there are three positively and three negatively charged residues, as reported above. Even in these complexes interactions are stabilized by many aromatic residues (see Table 2). This highlights that the structural determinants (in terms of charged and aromatic residues) of these complexes are quite conserved between PHO36 and ADIPOR1 and that osmotin interacts with ADIPOR1 even more than PHO36, as shown from a higher number of salt bridges (14 in Osmotin/ADIPOR1 and 5 in Osmotin/PHO36). In fact, osmotin shows for ADIPOR1 the highest affinity in terms of binding energy (see Figure S8) as well as the highest value of interface surface area (Å^2^) and of interaction residues.

All these results evidence that: i) the interaction regions are conserved in the complexes, ii) the interactions are on electrostatic basis but stabilized also by stacking interactions and iii) these findings can be used for the development of new ADIPORs agonists able to stimulate adiponectin receptors.

Since no therapy has yet been developed with adiponectin, being a novel hormone, we suggest that the osmotin could be used as a drug design template in therapeutic strategies for the treatment of obesity-related insulin resistance. Therefore, we have focused our attention on the osmotin peptide (sequence segment 157–165) that was observed at the interface between osmotin and ADIPOR1 as well as between osmotin and PHO36 (Figure S2 and Table 2). This peptide, also indicated as Peptide^OSM^, is stabilized from a disulphide bond that makes rigid its conformation. In fact, after molecular dynamics simulation, only the C-terminal residues of this peptide have been subjected to conformational changes (Figure S3). In the Peptide^OSM^/ADIPOR1 complex, the receptor exposes the same loops and the same positively charged residues (i.e. Lys 170 and Arg 235) evidenced in the other two complexes, i.e., ADIPOQ/ADIPOR1 and Osmotin/ADIPOR1 (Table 2). We have also modeled the complex between Peptide^OSM^ and PHO36, where we have found that the receptor exposes only Lys 176 whereas in the Osmotin/PHO36 complex also Glu 175 was involved in an interaction at the interface. However, both the Peptide^OSM^ and the whole osmotin show higher affinity for ADIPOR1 than for PHO36 (see Figure S8) with higher values of interface surface area (Å^2^), of number of interaction residues, of H-bonds, and of salt bridges, too (see Tables 1 and 2).

To support the conclusion reached by our *in silico* modeling, we have purified osmotin and synthesized the Peptide^OSM^. Afterward we have compared the *in vivo* effect of adiponectin, osmotin and Peptide^OSM^ on cellular cultures of synovial fibroblasts which are known to be specifically stimulated through the signaling downstream pathway of the ADIPOR1 receptor each time it is bound to the adiponectin. Our *in vivo* results demonstrate that both osmotin and Peptide^OSM^ interact with this same receptor by triggering the same specific pathway of adiponectin with the same final products, i.e., IL-6, TNF-alpha and IL-1beta, as already shown by Tang et al. (2007) [36]. The consideration that osmotin and adiponectin show related folds, although they do not share similar sequences, and that their similar structural behaviour *in silico* shows similar cellular effects *in vivo*, is surprising because, in general, an interaction requires that interacting residues be in clearly complementary sequence positions. This requires that the two structures must also predispose these interacting residues at a suitable distance and well-placed for an efficient interaction. In this case we have two similarly structured proteins with different sequences that interact with the same region of a third part (ADIPOR1) by triggering the same cellular process. Osmotin is not evolutively adapted to interact with ADIPOR1, therefore, we have only two choices: a) the osmotin structure is so flexible to adapt its sequence to the same ADIPOR1 region where adiponectin interacts too; b) the outer ADIPOR1 region is so flexible to adapt itself to different ligands structurally similar. Answer and possible explanation can be given on the basis of some of our recent observations. In fact, we have found that the outer loops and/or segments of numerous cytokine receptors have shown, by the consensus of eight different predictors and charge-hydrophobicity graphs, a consistent and abundant presence of intrinsically disordered structural stretches [41]. This finding opens a gleam on the binding ability of these receptors to different ligands [42] because of their very flexible and charged structures very prone to adapt to various structurally unrelated ligands. This idea suggests that the pleiotropy often attributed to cytokine receptors may be not always caused by a defective receptor that loses or misinterprets a signal but also by receptors that may have multiple ligands, although structurally unrelated, because of their peculiar structural properties due to the presence of intrinsically unordered regions. Because the peptide^OSM^ is structurally stable and structurally rather rigid with a good affinity to the ADIPOR1 receptor and correctly located to mimic the adiponectin binding, at the moment it represents, at best of our knowledge, the only good model of adiponectin agonist in vitro. Another similarly located peptide from adiponectin resulted very flexible and thus with lower affinity (*data not shown*).

Moreover, the peptide^OSM^ is quite conserved in the family of the thaumatin-like proteins; in fact, its sequence has an identity percentage of about 73% in respect to thaumatin-like proteins of other species (i.e. *Cicer aretinum*, *Vitis Vinifera*, *Ficus penile*, *Olea europaea*, etc.). In the literature no mention was found about an effect on ADIPOR1 exerted by other members of thaumatin-like proteins. However, a comparative analysis should be made on these proteins to study their putative role on the binding with ADIPOR1 but this is outside the aim of this paper.

In this work, we suggest that the Peptide^OSM^ could be used, opportunely modified to overcome difficulties in generating biologically active molecules, as a drug design template for the treatment of obesity-related insulin resistance. However, this study also raises questions about the role of this plant protein for the human health. No studies have been performed to assess if and how the osmotin affects the human health. This protein is enough stable and resistant to acidity and enzymes and thus might circulate through the body as peptides and/or interacting molecule by considering that it is present in many edible fruits and vegetables. In general, we have only a poor knowledge of the biologically active compounds in the foods. Our finding that an osmotin peptide, structurally very stable, is able to mimic the adiponectin, raises the question whether the osmotin could play any role in the health benefits attributed to diets high in fruits and vegetables. However this is another problem that will need different approaches to be studied.

## Supporting Information

Figure S1 3D model of two complexes. (A) Osmotin/PHO36 complex with Osmotin in fuchsia and PHO36 in cyan and (B) PeptideOSM/PHO36 complex with peptide in green and PHO36 in cyan.
(DOC)

Figure S2 Osmotin sequence. Amino acids in the helices are reported in grey but those in beta-strands are underlined. Interaction residues with the ADIPOR1 are evidenced in bold.
(DOC)

Figure S3 RMSD evolution during the molecular dynamics performed on the osmotin peptide (region 157-165).
(DOC)

Figure S4 Pairwise alignment between the sequences of human adiponectin and yeast Pho36 receptors.
(DOC)

Figure S5 Structure of plant osmotin (A) and human adiponectin (B). We have evidenced with a circle the domain I of the osmotin having a similar fold to that of the adiponectin.
(DOC)

Figure S6 Comparison between ADIPOQ/ADIPOR1 and osmotin/ADIPOR1 complexes concerning ADIPOR1 receptors superimposed. 3D model of Osmotin is colored in fuchsia and monomer A, B an C of ADIPOQ in red, blu and green, respectively.
(DOC)

Figure S7 Comparison of the osmotin and adiponectin residues which contact ADIPOR1 obtained superimposing ADIPOR1 receptors in two ADIPOQ/ADIPOR1 and osmotin/ADIPOR1 complexes. In particular, the residues are reported in CPK. Osmotin residues are colored in cyan but those in the adiponectin trimer in yellow.
(DOC)

Figure S8 Binding free energies for five complexes. The bars represent the binding energies (expressed in kcal/mol).
(DOC)

Table S1 Evaluation of Z-score by ProsaII program (A) and % residues in favored regions (B) for three adiponectin monomers in human and mouse.
(DOC)

Table S2 Analysis of the interaction among monomers in human and murine adiponectin trimers. The table shows interface accessible surface areas (Å^2^), number of interchain H-bonds, interaction residues and salt-bridges.
(DOC)
