# Correction: Structural and Functional Similarities between Osmotin from *Nicotiana Tabacum* Seeds and Human Adiponectin

**DOI:** 10.1371/annotation/69357261-7e31-40e0-96ff-13cdc783c768

**Published:** 2011-02-11

**Authors:** Marco Miele, Susan Costantini, Giovanni Colonna

The affiliation for the third author is incorrectly assigned. The correct affiliation for Giovanni Colonna is the first affiliation: Department of Biochemistry and Biophysics and CRISCEB - (Interdepartmental Research Center for Computational and Biotechnological Sciences), Second University of Naples, Naples, Italy. 

