# Supplementary material for: Structural and Functional Similarities between Osmotin from Nicotiana Tabacum Seeds and Human Adiponectin
Source: PLoS One. 2011 Feb 2;6(2):e16690. doi: 10.1371/journal.pone.0016690 (PMC3032776; doi:10.1371/journal.pone.0016690)
Supplement: Figure S1 — 3D model of two complexes. (A) Osmotin/PHO36 complex with Osmotin in fuchsia and PHO36 in cyan and (B) PeptideOSM/PHO36 complex with peptide in green and PHO36 in cyan. (DOC) [file pone.0016690.s001.doc]

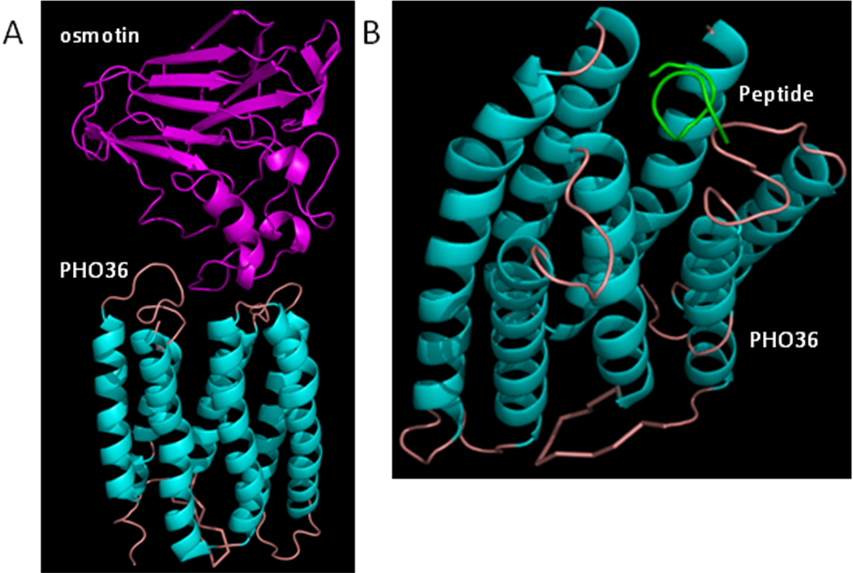


**Figure S1.** 3D model of two complexes. (A) Osmotin/PHO36 complex with Osmotin in fuchsia and PHO36 in cyan and (B) PeptideOSM/PHO36 complex with peptide in green and PHO36 in cyan
