# Supplementary material for: Structural and Functional Similarities between Osmotin from Nicotiana Tabacum Seeds and Human Adiponectin
Source: PLoS One. 2011 Feb 2;6(2):e16690. doi: 10.1371/journal.pone.0016690 (PMC3032776; doi:10.1371/journal.pone.0016690)
Supplement: Figure S2 — Osmotin sequence. Amino acids in the helices are reported in grey but those in beta-strands are underlined. Interaction residues with the ADIPOR1 are evidenced in bold. (DOC) [file pone.0016690.s002.doc]

ATIEVRNNCPYTVWAASTPIGGGRRLDRGQTWVINAPRGTKMARVWGRTNCNFNAAGRGT

CQTGDCGGVLQCTGWGKPPNTLAEYALDQFSGLDFWDISLVDGFNIPMTFAPTNPSG**GK**C

HAIHCTANINGECPRELRVPGGCNNPCTTFGGQQYC**CTQGPCGPTF**FS**K**FF**KQ**RCP**D**A**Y**S

**YP**Q**DDPT**S**TFT**CPGGSTNYRVIFCP

**Figure S2** Osmotin sequence. Amino acids in the helices are reported in grey but those in β-strands are underlined. Interaction residues with the ADIPOR1 are evidenced in bold.
