# Supplementary material for: Structural and Functional Similarities between Osmotin from Nicotiana Tabacum Seeds and Human Adiponectin
Source: PLoS One. 2011 Feb 2;6(2):e16690. doi: 10.1371/journal.pone.0016690 (PMC3032776; doi:10.1371/journal.pone.0016690)
Supplement: Figure S3 — RMSD evolution during the molecular dynamics performed on the osmotin peptide (region 157-165). (DOC) [file pone.0016690.s003.doc]

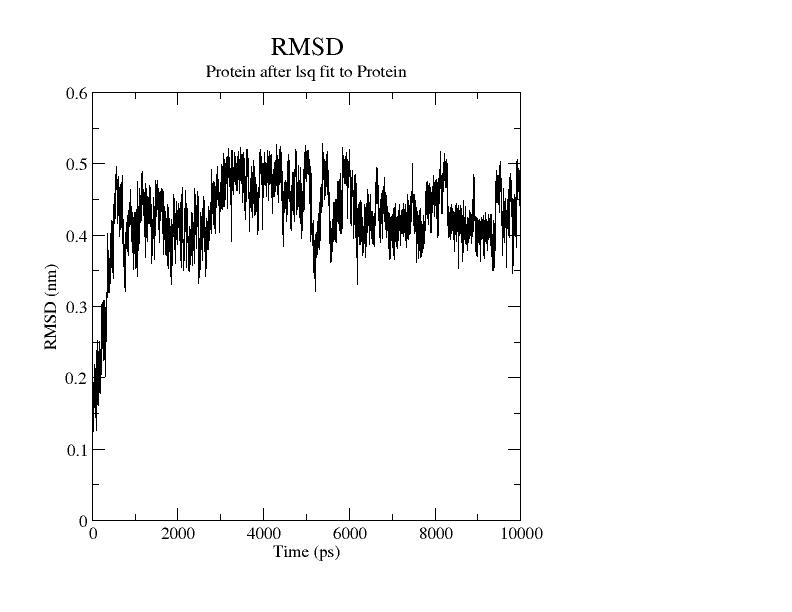


**Figure S3.** RMSD evolution during the molecular dynamics performed on the osmotin peptide (region 157-165).
