# Supplementary material for: Structural and Functional Similarities between Osmotin from Nicotiana Tabacum Seeds and Human Adiponectin
Source: PLoS One. 2011 Feb 2;6(2):e16690. doi: 10.1371/journal.pone.0016690 (PMC3032776; doi:10.1371/journal.pone.0016690)
Supplement: Figure S4 — Pairwise alignment between the sequences of human adiponectin and yeast Pho36 receptors. (DOC) [file pone.0016690.s004.doc]

ADR1_HUMAN MSSHKGSVVAQGNGAPASNREADTVELAELGPLLEEKGKRVIANPPKAEEEQTCPVPQEE

IZH2_YEAST -----------------------------MSTLLER--TKSVQELKKRAAGKTSANPAEV

:..***. .: : : * :*.. * *

ADR1_HUMAN EEEVRVLTLPLQAHHAMEKMEEFVYKVWEGRWRVIPYDVLPDWLKDNDYLLHGHRPPMPS

IZH2_YEAST AKAKKVLR------------------------RLYSWDEIPEWQRDNDFILHGYVKETSS

: :** *: .:* :*:* :***::***: .*

ADR1_HUMAN FRACFKSIFRIHTETGNIWTHLLGFVLFLFLGILTMLRPNMYFMAPLQEKVVFGMFFLGA

IZH2_YEAST FIETFKSLFYLHNESVNIYSHLIPALGFFTVLLLDKSTIKVFATTTWLDHMVIDLFYSGA

* ***:* :*.*: **::**: : *: : :* ::: :. :::*:.:*: **

ADR1_HUMAN VLCLSFSWLFHTVYCHSEKVSRTFSKLDYSGIALLIMGSFVPWLYYSFYCSPQPRLIYLS

IZH2_YEAST FACLILSSSFHCLKSHSLRIATLGNKLDYLGICILIVTSMVSILYYGYFEKFSLFCLFAL

. ** :* ** : .** ::: .**** **.:**: *:*. ***.:: . . ::

ADR1_HUMAN IVCVLGISAIIVAQWDRFATPKHRQTRAGVFLGLGLSGVVPTMHFTIAEGFVKATTVGQM

IZH2_YEAST ITVSFGIACSIVSLKDKFRKREWRPYRAGLFVCFGLSSIIPIFSGLYCYSFSEIWTQIQL

*. :**:. **: *:* . : * ***:*: :***.::* : . .* : * *:

ADR1_HUMAN GWFFLMAVMYITGAGLYAARIPERFFPGKFDIWFQSHQIFHVLVVAAAFVHFYGVSN-LQ

IZH2_YEAST FWVLLGGVLYIIGAVLYGMRFPEKICPGKFDIWGHSHQLFHFLVVIAALCHLRGLLNSYE

*.:* .*:** ** **. *:**:: ******* :***:**.*** **: *: *: * :

ADR1_HUMAN EFRYGLEGGCTDDTLL

IZH2_YEAST LVHIKMENGIVS----

.: :*.* ..

**Figure S4.** Pairwise alignment between the sequences of human adiponectin and yeast Pho36 receptors.
