# Supplementary material for: Structural and Functional Similarities between Osmotin from Nicotiana Tabacum Seeds and Human Adiponectin
Source: PLoS One. 2011 Feb 2;6(2):e16690. doi: 10.1371/journal.pone.0016690 (PMC3032776; doi:10.1371/journal.pone.0016690)
Supplement: Figure S5 — Structure of plant osmotin (A) and human adiponectin (B). We have evidenced with a circle the domain I of the osmotin having a similar fold to that of the adiponectin. (DOC) [file pone.0016690.s005.doc]

**A B**


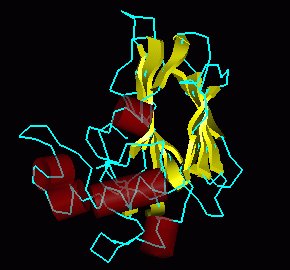

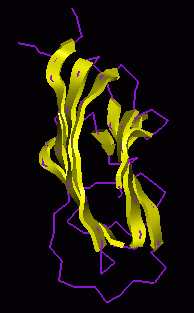


**Figure S5.** Structure of plant osmotin (A) and human adiponectin (B). We have evidenced with a circle the domain I of the osmotin having a similar fold to that of the adiponectin.
