# Supplementary material for: Structural and Functional Similarities between Osmotin from Nicotiana Tabacum Seeds and Human Adiponectin
Source: PLoS One. 2011 Feb 2;6(2):e16690. doi: 10.1371/journal.pone.0016690 (PMC3032776; doi:10.1371/journal.pone.0016690)
Supplement: Figure S6 — Comparison between ADIPOQ/ADIPOR1 and osmotin/ADIPOR1 complexes concerning ADIPOR1 receptors superimposed. 3D model of Osmotin is colored in fuchsia and monomer A, B an C of ADIPOQ in red, blu and green, respectively. (DOC) [file pone.0016690.s006.doc]

**
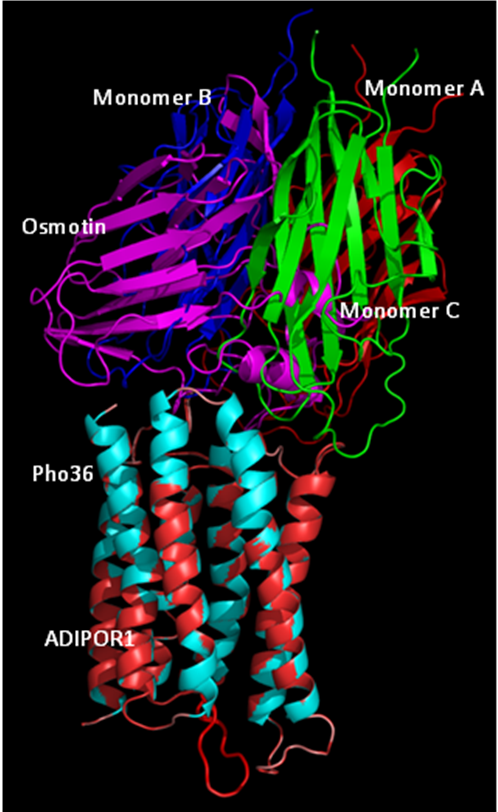
**



**Figure S6.** Comparison between ADIPOQ/ADIPOR1 and osmotin/ADIPOR1 complexes concerning ADIPOR1 receptors superimposed. 3D model of Osmotin is colored in fuchsia and monomer A, B an C of ADIPOQ in red, blu and green, respectively.
