# Supplementary material for: Structural and Functional Similarities between Osmotin from Nicotiana Tabacum Seeds and Human Adiponectin
Source: PLoS One. 2011 Feb 2;6(2):e16690. doi: 10.1371/journal.pone.0016690 (PMC3032776; doi:10.1371/journal.pone.0016690)
Supplement: Figure S7 — Comparison of the osmotin and adiponectin residues which contact ADIPOR1 obtained superimposing ADIPOR1 receptors in two ADIPOQ/ADIPOR1 and osmotin/ADIPOR1 complexes. In particular, the residues are reported in CPK. Osmotin residues are colored in cyan but those in the adiponectin trimer in yellow. (DOC) [file pone.0016690.s007.doc]

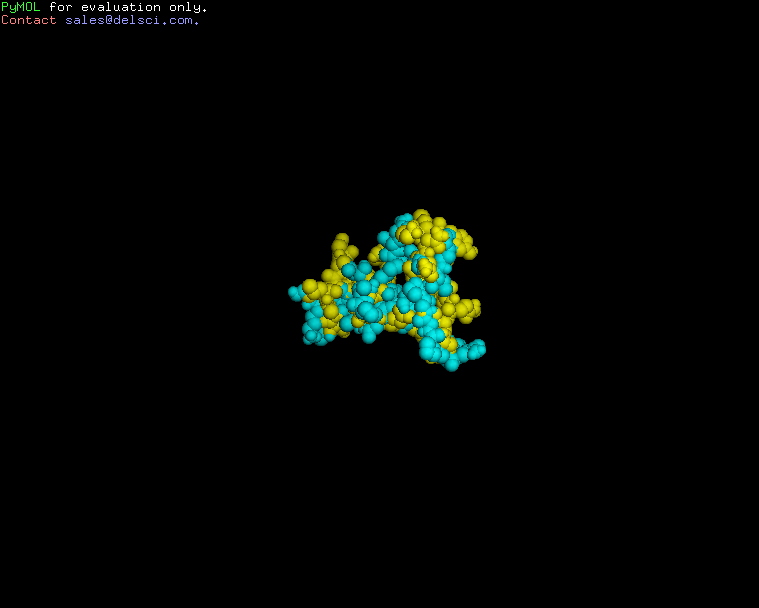


**Figure S7.** Comparison of the osmotin and adiponectin residues which contact ADIPOR1 obtained superimposing ADIPOR1 receptors in two ADIPOQ/ADIPOR1 and osmotin/ADIPOR1 complexes. In particular, the residues are reported in CPK. Osmotin residues are colored in cyan but those in the adiponectin trimer in yellow.
