# Supplementary material for: Structural and Functional Similarities between Osmotin from Nicotiana Tabacum Seeds and Human Adiponectin
Source: PLoS One. 2011 Feb 2;6(2):e16690. doi: 10.1371/journal.pone.0016690 (PMC3032776; doi:10.1371/journal.pone.0016690)
Supplement: Figure S8 — Binding free energies for five complexes. The bars represent the binding energies (expressed in kcal/mol). (DOC) [file pone.0016690.s008.doc]

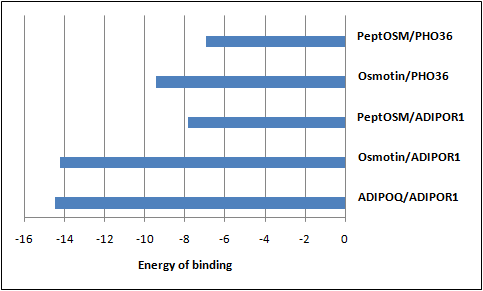


**Figure S8.** Binding free energies for five complexes. The bars represent the binding energies (expressed in kcal/mol).
