# Supplementary material for: Structural and Functional Similarities between Osmotin from Nicotiana Tabacum Seeds and Human Adiponectin
Source: PLoS One. 2011 Feb 2;6(2):e16690. doi: 10.1371/journal.pone.0016690 (PMC3032776; doi:10.1371/journal.pone.0016690)
Supplement: Table S1 — Evaluation of Z-score by ProsaII program (A) and % residues in favored regions (B) for three adiponectin monomers in human and mouse. (DOC) [file pone.0016690.s009.doc]

**Table S1.** Evaluation of Z-score by ProsaII program (A) and % residues in favored regions (B) for three adiponectin monomers in human and mouse.

A

|  | **Mouse** | **Human** |
| --- | --- | --- |
| **Chain A** | -4.32 | -4.67 |
| **Chain B** | -4.44 | -4.82 |
| **Chain C** | -4.34 | -4.59 |

B

|  | **Mouse** | **Human** |  |
| --- | --- | --- | --- |
| **Chain A** | 84.4 % | 92.6% | |
| **Chain B** | 85.2 % | 91.8% | |
| **Chain C** | 82.2 % | 91.9% | |
