# Supplementary material for: Structural and Functional Similarities between Osmotin from Nicotiana Tabacum Seeds and Human Adiponectin
Source: PLoS One. 2011 Feb 2;6(2):e16690. doi: 10.1371/journal.pone.0016690 (PMC3032776; doi:10.1371/journal.pone.0016690)
Supplement: Table S2 — Analysis of the interaction among monomers in human and murine adiponectin trimers. The table shows interface accessible surface areas (Å2), number of interchain H-bonds, interaction residues and salt-bridges. (DOC) [file pone.0016690.s010.doc]

**Table S2.** Analysis of the interaction among monomers in human and murine adiponectin trimers. The table shows interface accessible surface areas (Å^2^), number of interchain H-bonds, interaction residues and salt-bridges.

| **Chains** | **Interface Accessible Surface Area (Å^2^)** | **Residues in Interface** | **Hydrogen Bonds** | **Salt Bridges** |
| --- | --- | --- | --- | --- |
| Monomer A => Monomer B |  |  |  |  |
| Human | 1016.87 | 35 | 11 | 11 |
| Mouse | 906.33 | 29 | 12 | 13 |
| Monomer B => Monomer A |  |  |  |  |
| Human | 1088.80 | 26 | 11 | 12 |
| Mouse | 925.83 | 25 | 12 | 14 |
| Monomer A => Monomer C |  |  |  |  |
| Human | 1054.38 | 23 | 9 | 11 |
| Mouse | 925.23 | 25 | 13 | 13 |
| Monomer C => Monomer A |  |  |  |  |
| Human | 923.01 | 34 | 9 | 9 |
| Mouse | 893.70 | 31 | 13 | 13 |
| Monomer B => Monomer C |  |  |  |  |
| Human | 1000.70 | 35 | 8 | 12 |
| Mouse | 935.55 | 34 | 12 | 14 |
| Monomer C => monomer B |  |  |  |  |
| Human | 1107.50 | 24 | 8 | 9 |
| Mouse | 948.72 | 26 | 12 | 13 |
